# Single Residue Mutation in Active Site of Serine Acetyltransferase Isoform 3 from *Entamoeba histolytica* Assists in Partial Regaining of Feedback Inhibition by Cysteine

**DOI:** 10.1371/journal.pone.0055932

**Published:** 2013-02-21

**Authors:** Sudhir Kumar, Mohit Mazumder, Sudhaker Dharavath, S. Gourinath

**Affiliations:** School of Life Sciences, Jawaharlal Nehru University, New Delhi, Delhi, India; University of Hyderabad, India

## Abstract

The cysteine biosynthetic pathway is essential for survival of the protist pathogen *Entamoeba histolytica,* and functions by producing cysteine for countering oxidative attack during infection in human hosts. Serine acetyltransferase (SAT) and O-acetylserine sulfhydrylase (OASS) are involved in cysteine biosynthesis and are present in three isoforms each. While EhSAT1 and EhSAT2 are feedback inhibited by end product cysteine, EhSAT3 is nearly insensitive to such inhibition. The active site residues of EhSAT1 and of EhSAT3 are identical except for position 208, which is a histidine residue in EhSAT1 and a serine residue in EhSAT3. A combination of comparative modeling, multiple molecular dynamics simulations and free energy calculation studies showed a difference in binding energies of native EhSAT3 and of a S208H-EhSAT3 mutant for cysteine. Mutants have also been generated *in vitro*, replacing serine with histidine at position 208 in EhSAT3 and replacing histidine 208 with serine in EhSAT1. These mutants showed decreased affinity for substrate serine, as indicated by K_m_, compared to the native enzymes. Inhibition kinetics in the presence of physiological concentrations of serine show that IC50 of EhSAT1 increases by about 18 folds from 9.59 µM for native to 169.88 µM for H208S-EhSAT1 mutant. Similar measurements with EhSAT3 confirm it to be insensitive to cysteine inhibition while its mutant (S208H-EhSAT3) shows a gain of cysteine inhibition by 36% and the IC50 of 3.5 mM. Histidine 208 appears to be one of the important residues that distinguish the serine substrate from the cysteine inhibitor.

## Introduction

Serine acetyltransferase (SAT) (EC 2.3.1.30) which is the first member of the two-step cysteine biosynthetic pathway, catalyzes the formation of O-acetylserine (OAS) by transferring the acetyl group of acetyl Coenzyme A to serine (Ser) [1]. The SAT structure includes, in its C-terminal domain, a well conserved pair of so-called left handed parallel β-sheet helices (LβH), which arise due to a repeat sequence of [LIV]-[GAED]-X2-[STAV]-X [2] and also contribute to the formation of the active site. Comparison of the SAT structures available in native as well substrate/inhibitor bound forms shows that the residues involved in the substrate binding are highly conserved. SAT in bacteria and plants combines with the second member of the cysteine biosynthetic pathway, O-Acetyl Serine Sulfhydrylase (OASS) to form a cysteine synthase (CS) complex [3]. CS complex formation, which is favored when sufficient sulfur is available, is a part of the regulatory mechanism of the pathway where the activity of SAT increases and that of OASS decreases. Decrease in sulfide levels and excess production of OAS result in dissociation of the CS complex and an increase in OASS activity. Another level of regulation results from feedback inhibition of SAT by the cysteine (Cys) end product. In all of the organisms, where this pathway has been explored, most of the SAT isoforms are known to be competitively inhibited by cysteine, while a few SAT isoforms were also reported to exhibit a loss of inhibition by cysteine [3], [4], [5]. CS complex formation is absent in *E. histolytica,* and the feedback inhibition seems to be the only regulatory pathway in the protist pathogen. *E. histolytica* thus appears to be solely dependent on cysteine for anti-oxidative defense [6], [7], [8].

There are three isoforms of SAT in *Entamoeba histolytica*. EhSAT2 and EhSAT3 share 73% and 48% sequence identity respectively with EhSAT1 [4]. EhSAT1 was first characterized in *Entamoeba* by Nozaki and colleagues and they proposed the loss of interaction between SAT and OASS [8]. Hussain and colleagues characterized the remaining two isoforms and showed that feedback inhibition by Cys is different for all the three EhSAT isoforms. The EhSAT1 and EhSAT2 isoforms were inhibited by about 95% and 75% respectively, but EhSAT3 remained insensitive to cysteine even at high concentrations and in the presence of physiological concentrations of serine (3 mM) [4]. The crystal structure of EhSAT1 reported by our group, established the trimeric nature of EhSAT1 and proposed the reasons for loss of interaction between EhSAT and OASS [6]. The strength of SAT-OASS interactions seems to be dependent on the type of residue located at the C-terminal end of SAT and the extent to which the OASS active site cleft is opening [9].

Variants of SAT insensitive to cysteine inhibition are of major interest in industry where there is a need to produce L-cysteine at a large scale [10]. A M256I mutation in *E. coli* SAT resulted in excretion of cysteine upto 30 mg/l and desensitized the enzyme to cysteine feedback inhibition by 10 fold [10], [11]. M201V and E166G mutations in SAT render *E. coli* insensitive to cysteine inhibition [12].

**Figure 1:**
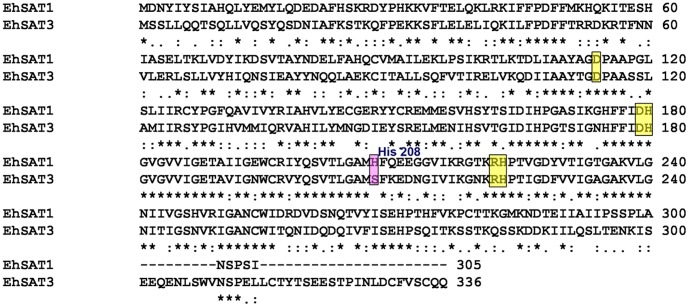
Protein sequence alignment of EhSAT1 and EhSAT3. Protein sequence alignment was done using ClustalW alignment program. Conserved active site residues are highlighted in yellow boxes while the active site residues (at position 208) that differ in the isoforms are highlighted in a pink box.

The active site (serine binding) residues, including three histidine residues are well conserved in all SATs. When we compared the sequence of EhSAT1 and EhSAT3, however, we found that histidine at position 208 is replaced with serine in EhSAT3 (Figure 1). The function of active site His residues has been investigated by Guan and coworkers in *H*. *influenza* [13]. The mutants generated for His154, His189 and Asp139 were checked for substrate binding and activity and it was seen that His189 could serve as an alternate base for catalysis in the absence of His154, while Asp139 increases the basicity of His154 for electron transfer [13]. All of these residues are conserved in EhSAT1 and EhSAT3 and they correspond, respectively, to His180, His223, and Asp164 in *Entamoeba histolytica*. So far, no studies have been carried out on the residues corresponding to EhSAT His208.

**Figure 2:**
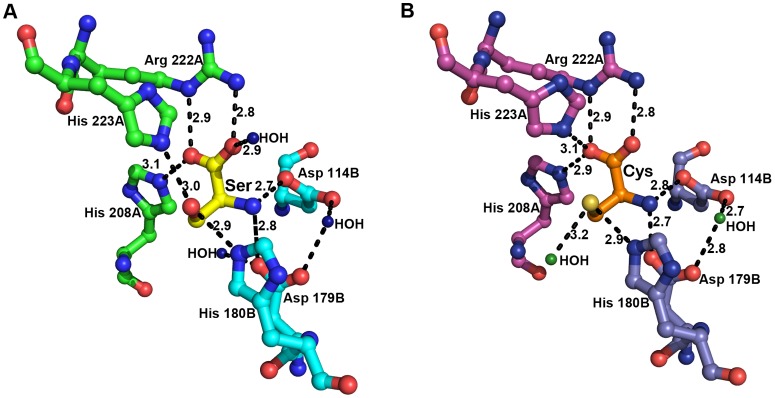
Active site interactions of EhSAT1. Image showing interaction of A) L-ser and B) L-Cys with the active site residues of EhSAT1 as visualized in crystal structure of EhSAT1-ser (PDB id 3Q1X) and EhSAT1-cys (PDB id 3P47) [8]. A) A salt bridge is formed in between the carboxyl group of Ser and the side chain of Arg 222A. This carboxyl group also makes a hydrogen bond with side chain of His 208A and a water molecule. Side chains of His 223A and His 180B make other hydrogen bonds with the hydroxyl group of the serine. The amino group of serine forms a salt bridge with the carboxyl group of the Asp 114B and Asp 179B. B) Interactions with the inhibitor L-cysteine bound at active site of EhSAT1. Cysteine coordinates with the same residues as that of serine, but with minor repositioning. The carboxyl oxygen interacts with the amide group of the Arg 222A and also with His 223A and His 208A. The thiol group interacts with His 180B, His 223A, and a water molecule. The amine of cysteine bonds with the carboxyl groups of the Asp 114B and Asp 179B. The image was prepared using Pymol [26].

In EhSAT1, His208 is in close proximity to the sulfur of bound cysteine, and its orientation differs from that in the serine-bound structure. This His208 movement appears to cause a change in the orientation of His223, and thus of a couple of hydrogen bonds as well (Figure 2). The equivalent residue of His208 in EhSAT3 is serine, where the feedback inhibition is lost. We expected that replacement of His with ser at position 208 may play a role in the loss of sensitivity to cysteine. Here, in this study, we investigate the role of serine/histidine at this position in the active site and the effect on Cys inhibition in both EhSAT1 and EhSAT3.

We have used both multiple molecular dynamics simulations and kinetic studies to determine the functional role of residue 208 by comparing native and mutated forms of both EhSAT1 and EHSAT3. Binding free energies have been calculated for native EhSAT1 and His208Ser mutant of EhSAT1 (denoted as H208S-EhSAT1 hereafter) with substrate Ser and inhibitor Cys. Similarly, after the EhSAT3 model was built using EhSAT1 structure as a model, binding energies were calculated after 10 ns simulations for EhSAT3 and for Ser208His mutant (denoted as S208H-EhSAT3 hereafter) with substrate Ser and inhibitor Cys. PCR based site directed mutagenesis was used to make the above mentioned mutants and enzyme kinetics and inhibition kinetics were compared for both native and mutant enzyme. The native EhSAT1 was 97% inhibited with 0.1 mM Cys, while the H208S-EhSAT1 was only 85% inhibited, even at 2 mM cysteine concentration. While native EhSAT3 was hardly inhibited, the S208H-EhSAT3 was inhibited by about 30%. Both the *in silico* and *in vitro* results indicate that the identity of side chain (Ser/His) at position 208 plays a crucial role in distinguishing bound substrate (serine) from inhibitor (cysteine), and hence in determining feedback inhibition.

## Materials and Methods

### Structural Modeling of EhSAT3

The sequence alignment of EhSAT1 and EhSAT3 revealed that the isoforms share 48% identity and 75% similarity, indicating that EhSAT1 is a suitable template for comparative modeling of EhSAT3. The initial three-dimensional model of EhSAT3 was built using the EhSAT1 structure (PDB id: 3P47) as a template and by using MODELLER9v9 [14]. The structures of EhSAT3 in complex with cysteine or serine were obtained by superimposing them on the EhSAT1 complex structures (PDB id: 3Q1X, 3P47). The best models were chosen on the basis of their lowest DOPE score and the highest GA341 assessment score and further validated by PROCHECK [15].

### *Insilico* Mutants

To evaluate the proposed mechanism of cysteine and serine binding to EhSAT1 and EhSAT3, two mutant constructs were prepared using the rotamer library in CHIMERA [16]. Mutation of histidine 208 to serine was performed on the EhSAT1-serine and EhSAT1-cysteine complex crystal structures. The serine 208 was mutated to histidine in the homology model of EhSAT3 and its complexes.

### Molecular Dynamics (MD) Simulations

**Figure 3:**
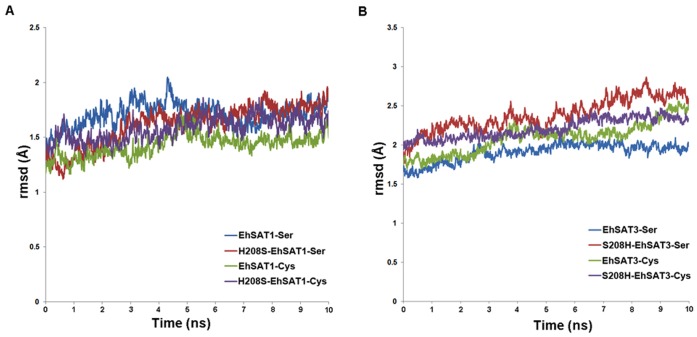
All atom root-mean-square deviations after performing 10 ns of molecular dynamics simulation of A) EhSAT1-cysteine & H208S-EhSAT1-cysteine, EhSAT1-serine & H208S-EhSAT1-serine B) EhSAT3-cysteine & S208H-EhSAT3-cysteine, EhSAT3-serine & S208H-EhSAT3-serine. The trend obtained from EhSAT1 & EhSAT3 showing slightly higher rmsd’s for the mutants compared to the native suggesting that the native forms are relatively more stable than the mutants.

We performed MD simulations using the AMBER 9.0 suite [17]. The AMBER99SB [18] force field was used to define potential and atom types for all calculations. The input files for energy minimization, dynamics and analysis were prepared with tleap, a program from the AMBER suite. Each system was solvated in an octahedron box of TIP3P water molecules with a margin of 12 Å along each dimension, followed by addition of Na^+^ ions to counteract the negative charge and neutralize the system. To equilibrate the solvated complex, a short energy minimization with restrained protein (1,000 steps, conjugate gradient) was first performed followed by 50 ps of heating from 0 to 300 K and 50 ps of density equilibration with weak restraints on the complex. This was then followed by a 500 ps of constant pressure equilibration without restrains at 300 K. After equilibration, production runs were performed with a total simulation length of 10 ns for all 8 complexes and the trajectories of the complex structure were recorded out every 10 ps (Figure 3). All simulations included the SHAKE algorithm to constrain the bonds to hydrogen atoms, Particle Mesh Ewald [19] with default parameters and an 8 Å cutoff. The calculations for all the eight systems (EhSAT1, EhSAT3 and their mutants in complex with Ser and Cys) were set up using similar protocols. We chose to perform small time scale dynamics because the eight complexes studied were very large with more than 800 residues. We simulated each of the complexes for 10 ns.

### Determination of Binding Free Energy Using MM-PBSA Method

The binding free energies were calculated using the Molecular Mechanics-Poisson Boltzmann Surface Area (MM-PBSA) method [20] as implemented in AMBER 11 [17] using the Ambertools 1.4. The MM-PBSA [20] method computes the interaction energy and solvation free energy for the complex, receptor and ligand and averages the results to obtain an estimate of the binding free energy (Table 1). For the calculation of nonpolar contributions to the solvation free energy, solvent-accessible-surface-area-dependent terms were used, in which the surface area is computed with Paul Beroza’s molsurf program [21]. In this study, free energy calculations were conducted using 1000 frames collected during MD simulations of the EhSAT1 and EhSAT3 complexes.

**Table 1:** The binding free energy of EhSAT1 and EhSAT3 complexes.

Complex	ΔE _Ele_	ΔE _Vdw_	ΔE_MM_	ΔG_sol-ele_	ΔG _sol-np_	ΔG_Polar_	ΔG_nonpolar_	ΔG _Bind_
**EhSAT1-Cys**	−41.11	−13.5	−54.61	24.77	−0.92	−16.33	−14.42	−30.75
**H208S-EhSAT1-Cys**	8.56	−13.26	−4.7	−7.92	−0.95	0.64	−14.21	−13.57
**EhSAT1-Ser**	−7.78	−9.43	−17.21	17.44	−0.83	9.66	−10.26	−0.6
**H208S-EhSAT1-Ser**	19.32	−11.27	8.05	−6.67	−0.8	12.65	−12.07	0.58
**EhSAT3-Cys**	34.59	−14.64	19.95	−13.63	−1.01	20.96	−15.64	5.32
**S208H-EhSAT3-Cys**	−48.29	−12.17	−60.46	40.17	−0.9	−8.12	−13.06	−21.19
**EhSAT3-Ser**	−27.72	−10.38	−38.1	38.37	−0.81	10.65	−11.19	−0.55
**S208H-EhSAT3-Ser**	−37.96	−13.28	−51.23	46.59	−0.64	8.63	−13.92	−5.29

The table shows the detailed contribution of energy components calculated using Poisson Boltzmann Surface Area (MM-PBSA) method for EhSAT1, EhSAT3 and their mutants to evaluate their binding activity. Here ΔE_Ele_, electrostatic interactions; ΔE_Vdw_, van der Waals interactions, ΔE_MM_ = ΔE _Ele_+ΔE_Vdw_, ΔG_sol-ele_: polar solvation free energy are calculated by solving the Poisson-Boltzmann equation PB; ΔG_sol-np_, non-polar solvation free energy, ΔG_polar_ = ΔE _Ele_+ΔG_sol-ele_; ΔG_nonpolar_ = ΔE_Vdw_+ΔG_sol-np_, ΔG_Bind_ = estimated total binding free energy.

### Per Residue Energy Contribution

**Figure 4:**
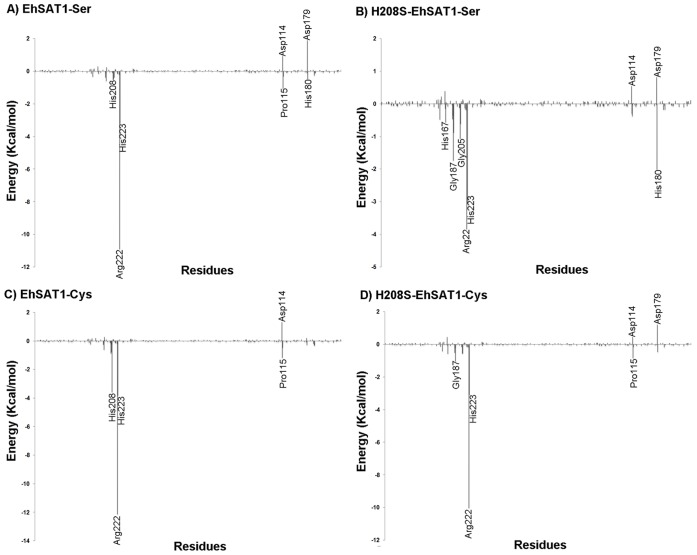
Per-residue energy contributions. The contribution of individual amino acid residues of EhSAT1 and H208S-EhSAT1 (which mimics the EhSAT3 active site) to the binding of serine and cysteine calculated using MM-GBSA method to find the important residues involved in specific binding shown in A) EhSAT1-Ser, B) H208S-EhSAT1-Ser, C) EhSAT1-Cys and D) H208S-EhSAT1-Cys. Arg 222 and His 223 provide the strongest contributions in all of the complexes, although the strength of the contributions decreases for the mutants. Strikingly, His208 contributes to binding affinity only for the EhSAT1-Cys complex structure, where it is the third strongest contributor. This contribution is not seen in EhSAT1-Ser or any of the mutant structures.

In order to obtain detailed insight into the contribution of each residue in the binding energy of serine and cysteine in EhSAT1, we used the Molecular Mechanics Generalized Born Surface Area method [20]. Here the interaction energies of each residue were calculated by using molecular mechanics and solvation energies without considering the contribution of entropies (Figure 4).

### Site Directed Mutagenesis

The PCR based site directed mutagenesis was carried out using the protocol of Edelheit *et al.* [22] to introduce a serine in place of histidine at position 208 of EhSAT1 and histidine in place of serine at position 208 of EhSAT3, using the primers listed in Table 2. The mutated triplet is shown in bold in all the primers. The mutations were confirmed by nucleotide sequencing (Figure S1).

**Table 2:** Primers used for Site directed Mutagenesis.

SAT3	
**P1**	5′ GGTGCAATG**CAT**TTCAAAGAA 3′
**P2**	3′ CCACGTTAC**GTA**AAGTTTCTT 5′
**SAT1**	
**P1**	5′ GGAGCAATG**AGT**TTCCAAGAG 3′
**P2**	3′ CCTCGTTAC**TCA**AAGGTTCTC 5′

### Expression and Purification

The method used for expression and purification of all the proteins used in this study is similar to that used in our previous paper [6]. The concentrated purified protein was checked for homogeneity on SDS-PAGE and stored at −80°C. The levels of expression of the native and mutant enzymes were similar. The protein purity and homogeneity was checked by SDS PAGE and gel filtration chromatography (Figure S2). Native and mutant EhSAT1 proteins were stable, while EhSAT3 and its mutant showed loss of activity due to aggregation over long storage at 4°C.

### Kinetics

**Figure 5:**
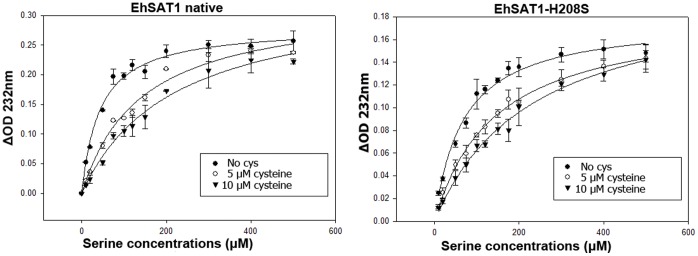
Kinetic study of EhSAT1. Michaelis Menten representation of velocity values plotted against the serine concentrations for EhSAT1 native and mutant (H208S). Kinetic studies were done in 50 mM Tris buffer pH 8.0 keeping acetyl CoA concentration constant at 0.1 mM and varying concentration of serine from 10 µM to 500 µM. The inhibition studies were done in presence of 5 µM and 10 µM of cysteine. K_m_ was calculated by the Michaelis Menten equation using Sigma Plot software. Standard deviations are calculated from the three independent experiments for each substrate concentration values. The competitive inhibition is intact in the both native as well as mutant but the overall activity of the mutant has decreased by about 50%.

**Figure 6:**
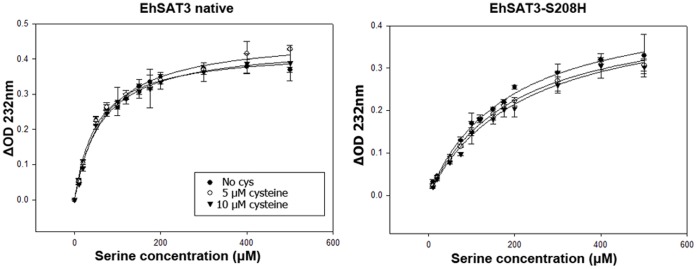
Kinetic study of EhSAT3. Michaelis Menten representation of velocity values plotted against the serine concentrations for EhSAT3 native and mutant (S208H). Kinetic studies were done in 50 mM Tris buffer pH 8.0 keeping acetyl CoA concentration constant at 0.1 mM and varying concentration of serine from 10 µM to 500 µM. The inhibition studies were done in presence of 5 µM and 10 µM of cysteine. K_m_ was calculated by the Michaelis Menten equation using Sigma Plot software. Standard deviations are calculated from the three independent experiments for each substrate concentration values. EhSAT3 activity was not affected by the mutation but the mutant EhSAT3 show increased sensitiveness to cysteine inhibition.

The SAT enzyme kinetics were performed using a protocol similar to that reported by Hussain and colleagues [4]. The reaction mixture contains 50 mM Tris pH 8.0, 0.1 mM acetyl coenzyme A and 2.5 µg enzyme. Each reaction was started by adding L-serine and the decrease in absorption was monitored at 232 nm due to cleavage of thioester bond in acetyl CoA. The reactions were carried out for 2 minutes each at room temperature and monitored at different serine concentrations ranging from 10 µM to 500 µM. Cysteine inhibition was measured at 5 and 10 µM. V_max_ and K_m_ values were calculated using the Michaelis Menten equation (Figure 5, 6; Table 3). All values are averages of at least 3 independent experiments.

**Table 3:** Comparison of K_m_ for both native and mutant proteins of EhSAT1 and EhSAT3 at different cysteine concentrations.

	K_m_ (µM)	K_m_ (µM) at 5 µM Cysteine	K_m_ (µM) at 10 µM Cysteine Cysteine10 µM cys
EhSAT1 Native	43.5±4.9	140.7±18.8	217.8±29.1
H208S-EhSAT1	68.4±6.8	142.4±8.1	232.1±22.8
EhSAT3 Native	52.0±4.7	62.7±4.5	67.3±6.9
S208H-EhSAT3	185.5±12.7	181.4±15.8	210.9±25.9

### Effect of Cysteine at Physiological Serine Concentrations

**Figure 7:**
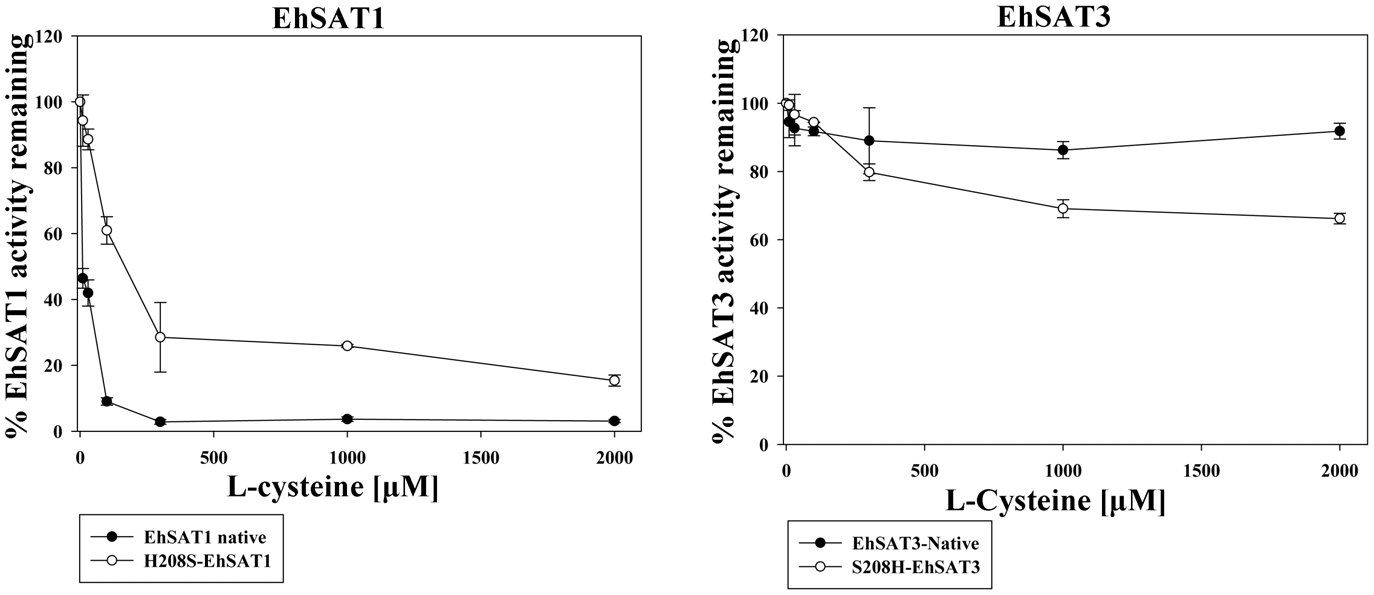
The percent of SAT activity remaining in the presence of increasing concentrations of cysteine. Reactions were carried out in the presence of 3 mM serine. Native EhSAT1 was inhibited completely by 0.1 mM cysteine while the H208S-EhSAT1 mutant was inhibited by only about 85% even at a concentration of 2 mM Cys. As expected, native EhSAT3 was not inhibited by cysteine but the S208H-EHSAT3 showed about 36% inhibition.

SAT activity was measured in the presence of 3 mM serine, which is equivalent to the concentration used in axenically cultured trophozoites of *E. histolytica* [23], to test the effect of cysteine on SAT. Inhibition values of all SAT constructs were measured while keeping the Ser concentration constant at 3 mM and varying the Cys concentration from 0 to 2 mM (Figure 7).

## Results

### Comparison of EhSAT1 and EhSAT3 Protein

Alignment of EhSAT1 and EhSAT3 protein sequences (Figure 1) reveals that position 208, which is a histidine residue in EhSAT1 and binds the serine/cysteine ligand, is a serine residue in EhSAT3. All other active site residues are conserved between both proteins. The loop region between β sheet helix 2 and 3, which contains position 208 and is close to the active site and to the C-terminal end, is the least conserved region of the protein.

### Modeling, Simulations and Free Energy Calculations for EhSATs

The EhSAT1-Ser/Cys structures were obtained from the RCSB and the structures of EhSAT3-ligand complexes were constructed by comparative modeling using EhSAT1 as a template. Four complexes of each protein were subjected to MD simulations to determine whether and how the binding of substrate and inhibitor change the flexibility, specificity and conformation of the enzyme.

### Effect of Mutation on EhSAT’s Overall Structure

We calculated the root-mean-square deviation (rmsd) of the backbone atoms of EhSAT1 and EhSAT3 complexes from their MD trajectories using the crystal structure and the model as the starting structure. The rmsd values for EhSAT1 and EhSAT3 simulations were plotted as a function of time for each of the complexes (Figure 3). As can be seen, the rmsd values for all the simulations were fairly low. The calculated average values of rmsd in EhSAT1 complexes (native & mutant) were less than 1.7 Å, obtained from 1000 snapshots suggesting the structural stability of both native and mutant form. Similarly in the modeled structure of EhSAT3, the average values were less than 2 Å in case of native and slightly above 2 Å in the mutants. All of the rmsd plots in which each frame was recorded every 10 ps indicated that the complexes are stable over the timescale of 10 ns (Figure 3).

### Effect of Mutation on EhSAT1 Substrate/inhibitor Binding Affinity

We have used the single trajectory approach to calculate the relative binding free energy and the contributions of various parameters such as van der Waals, non-polar, electrostatic energy and solvation free energy. The calculated energy components and binding free energy for all 8 systems are listed in Table 1. As calculated using the MM-PBSA method, the relative binding free energy (ΔG_bind_) between cysteine and EhSAT1 is −30.74 kcal/mol whereas the energy between H208S-EhSAT1 and cysteine is −13.57 kcal/mol. These differences in the binding free energy (ΔG_bind_) clearly suggest that the mutation reduces the affinity of EhSAT1 for cysteine, indicating the importance of His 208 in the binding pocket. Furthermore, differences were also calculated for serine binding; the binding free energy between native EhSAT1 and serine is −0.60 kcal/mol, whereas that between the H208S-EhSAT1 and serine is 0.58 kcal/mol. These calculations also suggest that cysteine binds both the native and mutant EhSAT1’s with higher affinity than does serine, although the difference in binding energy is very much less in the mutant protein. If we look into the binding modes of cysteine and serine in EhSAT1, the major difference in terms of individual energy contributions comes from the electrostatic interactions. The ΔE_ele_ for cysteine is −41.10 kcal/mol whereas for serine it is −7.77 kcal/mol. In all of the cysteine and serine bound systems and their H208S constructs, the major binding difference was from the electrostatic contribution to the solvation free energy as calculated by the molecular mechanic (MM) force field (ELE).

### Effect of Mutation on EhSAT3 Substrate/inhibitor Binding Affinity

A similar study was conducted on native EhSAT3 and S208H-EhSAT3. The calculated binding free energy between native EhSAT3 and cysteine is 5.32 kcal/mol, indicating low affinity, whereas the calculated binding free energy between the S208H-EhSAT3 and cysteine indicates a better affinity of −21.18 kcal/mol. The significance of histidine at position 208 for the binding of cysteine is thus indicated by calculations using EhSAT3 as well as EhSAT1. However, the calculated binding free energies of serine to native and S208H-EhSAT3 are −0.54 kcal/mol and −5.29 kcal/mol, respectively. Native EhSAT3 thus appears to have better binding affinity for Ser then Cys, indicating Cys should not show much inhibition. In contrast, S208H-EhSAT3 shows better affinity for Cys, indicating that Cys should inhibit more effectively here.

### Contribution of Hotspot and their Role in EhSAT1 Substrate/inhibitor Binding

After comparing the calculated binding free energies for the complexes, individual energy decompositions for all the residues in EhSAT1 were calculated to find the hotspots that play important roles in substrate and inhibitor binding. We also wanted to see the effect of H208S mutation and the difference it makes to the nearby residues in terms of binding energy. The per residue energy profiles for substrate serine and inhibitor cysteine are plotted in figure 4. Arg 222 has the highest binding free energy contribution in native EhSAT1-Cys and EhSAT1-Ser complex structures, at −12.14 kcal/mol and −10.91 kcal/mol, respectively. It is mainly driven by formation of salt bridge with the carboxyl terminal of the both cysteine and serine. His223 has the second highest contribution of binding free energy for both complexes. These results clearly suggest both of the residues play a similar role in substrate and inhibitor binding. In the cysteine bound structure, His208 has the third highest binding free energy contribution, at −3.6 kcal/mol. Interestingly in the serine bound structure, the contribution of His208 is much less, at −0.39 kcal/mol. This indicates that His208 plays a more important role in inhibitor binding than in substrate binding and might thus be involved in the selection of cysteine over serine.

Simulation studies were also carried out for the binding of serine and cysteine to H208S-EhSAT1, the mutant which mimics the EhSAT3 active site. These studies indicate that the contribution of residues 222 and 223 to the binding affinity, although higher than that of other residues, is decreased relative to their levels in the native EhSAT1 (Figure 4). Moreover, Ser208 provides no significant contribution to binding affinity, in contrast to the significant binding affinity calculated for His208 in the native enzyme. The replacement of His208 with Ser thus appears to diminish the overall affinity for substrate and inhibitor both directly and by affecting the affinity of nearby active site residues.

### Comparison of Kinetic Parameters

The reaction between native EhSAT1, acetyl coenzyme A and serine shows a K_m_ of 43.54 µM, which is a little different than that reported earlier by Hussain and colleagues [4]. In the presence of 5 and 10 µM cysteine, the K_m_ increases to 140.7 µM and 217.8 µM respectively (Figure 5; Table 3). The change is in agreement with the earlier published reports where it is known that cysteine competitively inhibits serine. In the case of the His208Ser- EhSAT1 mutant, there is a marked decrease in the activity of the enzyme and the K_m_ is increased to 68.4 µM. In presence of 5 and 10 µM of cysteine, the K_m_ increases to 142.4 and 232.1 µM respectively, indicating that the competitive inhibition is still intact.

For native EhSAT3, the K_m_ in the absence of cysteine (52.0 µM) remains nearly the same as that in the presence of cysteine (between 62.7 µM and 67.3 µM), consistent with a lack of competitive inhibition with Cys as previously observed for EhSAT3 [4]. For S208H-EhSAT3, the K_m_ in the absence of cysteine is 185.5 µM, which shows that the mutation has also affected serine binding (Figure 6, Table 3). There is an increase in K_m_ in the presence of 10 µM cysteine, showing the partial regain in sensitivity of the enzyme toward cysteine.

### Effect of Cysteine at Physiological Serine Concentration

3 mM serine is considered to be the physiological concentration of the axenically cultured amoebae [23]. Inhibition studies in the presence of this concentration of serine indicate that EhSAT1 was inhibited almost completely, with only ∼3% of EhSAT1 activity remaining at 300 µM cysteine. For His208Ser-EhSAT1, at a cysteine concentration of 2 mM, the enzyme was about 15% active. The IC50 of EhSAT1 increases by about 18 folds from 9.59 µM (for native) to 169.88 µM for H208S-EhSAT1. Similar measurements with EhSAT3 confirm it to be insensitive to cysteine inhibition while its mutant (S208H-EhSAT3) shows a gain of cysteine inhibition by 36% and the IC50 of 3.5 mM.

As described above, EhSAT3 is not inhibited by cysteine. Even at a very high cysteine concentration of 2 mM, EhSAT3 remains more than 90% active. The S208H-EhSAT3, however, does show some response to the cysteine inhibition; at 2 mM cysteine, the activity of mutated enzyme was inhibited by about 36% (Figure 7).

## Discussion

Of the three isoforms of SAT expressed by *E. histolytica*, it has been established that activity of EhSAT1 is modulated by the feedback inhibitor Cys, and that EhSAT3 is insensitive to (i.e., not inhibited by) Cys [4]. Moreover, previous structural and biochemical studies on EhSAT1 have shown that substrate Ser and inhibitor Cys bind to the same location in the active site [6]. In acetyltransferases, the imidazole side chain of the active site His residue acts as a general base in the acyltransferase reaction, and catalyzes the direct attack on the acetyl coenzyme A. This reaction seems to be disrupted by the binding of the cysteine. In these studies it was shown that there is a small change in the orientation of His208 when cysteine is bound compared to when serine is bound, due to the large size of cysteine’s sulfur. A sequence comparison of EhSAT1 and EhSAT3 shows that the equivalent residue of His208 in EhSAT1 is serine in EhSAT3. We speculated that replacement of histidine by serine in EhSAT3 may play a major role in loss of feedback inhibition by Cys. To test this hypothesis, both *in silico* and *in vitro* experiments were carried out.

A mutation of histidine208 to serine in EhSAT1 was designed *in silico*, followed by molecular dynamics simulation and MM-PBSA calculation of the free binding energy for both serine and cysteine. His 208 makes a hydrogen bond with carboxyl group of both Ser and Cysteine in their respective complex structures. The orientation of the His 208 and His 223 are different and hydrogen bonding pattern are also different when bound to Cys compared to Ser. When the His 208 is mutated to Ser in EhSAT1, according to the model, there are no interactions with Ser to bound amino acid (either Ser/Cys) bound in the active site and there is no structural change in orientation of His 223 (Figure S3). Furthermore, an EhSAT3 model was generated (based on the experimentally determined coordinates of EhSAT1), and then similar, *in silico* calculations on a mutation of serine208 to histidine in this model were carried out. Similar kinds of interactions are also observed EhSAT3. In the native EhSAT3, Ser 208 does not interact with bound Ser/Cys in the active site (Figure S3), while in mutant His 208-EhSAT3, the His residues forms hydrogen bond with carboxyl group of the bound amino acid (Figure S3). The binding energies of serine and cysteine were calculated after 10 ns simulations for both native and S208H-EhSAT3.

In the complexes of EhSAT1, cysteine showed a higher calculated binding free energy compared to serine. For the H208S-EhSAT1 mutant, the calculated binding energy of serine and of cysteine are both relatively low compared to that for the native enzyme, but the ΔG value for Cys remains comparatively better than for Ser. The binding energy difference between Cys and Ser, however, was narrowed in the mutant, indicating lower inhibition of the mutant by cysteine compared to the native enzyme. The reduction in the binding energy of H208S-EhSAT1 for Ser is indicated by lower activity of the mutant enzyme (Figure 5). The binding energy values of Cys for native EhSAT3 are positive, indicating poor binding affinity and no inhibition, while S208H-EhSAT3 showed favorable binding energy, indicating higher binding affinity and that the S208H-EhSAT3 should show feedback inhibition with Cys. The energy contribution of individual amino acid residues of EhSAT1 and H208S-EhSAT1 clearly showed the importance of His208 in the binding pocket for cysteine binding and its reduced involvement in serine binding, suggesting its possible role in selection of cysteine over serine.

To further validate the results obtained from MM-PBSA free energy calculations, mutant enzymes were expressed and kinetics was performed. Following the trend obtained from theoretical calculations, the *in vitro* experiments showed that activity of H208S-EhSAT1 (both K_m_ and k_cat_) was reduced and there was some difference in the feedback inhibition of cysteine. Although the mutation of EhSAT3 resulted in a loss of affinity for serine according to the increase in the value of K_m_, the value of kcat was similar to that of native EhSAT3. Even though there was inhibition of S208H-EhSAT3 with cysteine, the inhibition was only about 35% with 2 mM cysteine, which is very low compared to that of EhSAT1.

The experiments on the mutation of residue 208 at the active site clearly indicated that this residue does play an important role in recognition of the feedback inhibitor. The native EhSAT1 is inhibited almost 97% with 300 µM Cys, while H208S-EhSAT1 is inhibited only 85% even with 2 mM Cys concentration. Moreover, the S208H-EhSAT3 was inhibited 35% with 2 mM Cys, while native EhSAT3 was hardly inhibited (Figure 7). Even though the effect is not 100%, it is conclusive that this specific residue is one of the important residues in differentiating the inhibitor Cys and the substrate Ser.

The reaction kinetics is not only dependent on the final binding energies of substrate/inhibitor at the active site, but also on the transfer of substrate/inhibitor to the active site. His 208 is located in the long loop between coil 2 and coil 3 of the left handed β helix domain, where there are significant differences in the sequence of this long loop in EhSAT1 compared to EhSAT3 (Figure 1). Double mutation of M201V along with E166G in *E. coli* SAT renders the enzyme insensitive to cysteine inhibition [12]. In EhSAT3, the position equivalent to Met201 is already occupied by valine, which may account for its lack of sensitivity for cysteine inhibition. There are several studies indicating the importance of the C-terminal residues in Cys feedback inhibition [24], [25]. The Saito and his coworkers showed that Met to Ile mutation at the C-terminal 280 residue in the watermelon SAT decreased the Cys inhibition by 25 folds and Gly 277 Cys mutant also showed similar Cys inhibition [24]. In both EhSAT1 and EhSAT3, the equivalent residue of 280 is already Ile and 277 is Ile and Glu respectively (Figure S4), therefore we expect these residues may not play any role in Cys inhibition in Entamoeba SAT’s. By comparing *A. thaliana* and *T. goesingense* SAT sequences, GunNan and Salt [25] proposed that Pro and Ala at 268 and 270^th^ position of *T. goesingense* SAT might be responsible for Cys insensitivity. At these positions EhSAT1 has serine and glutanmine, while EhSAT3 has glutamine at both positions; moreover this C-terminal end region has very little sequence similarity between EhSAT’s and TgSAT (Figure S4). Therefore these residues may not play any role in the loss of feedback inhibition in EhSAT3.

The differences between SAT1 and SAT3 around the active site, involving the loop connecting β-coil 2 and β-coil 3 and C-terminal end, which is close to Acetyl Co-A binding site. As observed before and discussed above the C-terminal residues play an important role in Cys sensitivity. These regions may not be playing a direct role in the binding of the substrate or inhibitor, but appears to be affecting the path of the substrate/inhibitor before reaching the active site.

The structural and functional studies of SAT isoforms have revealed that *Entamoeba* has adopted survival strategies that bypass the regulation mechanism of cysteine biosynthesis [6]. Although the information regarding the localization or time of expression of SAT isoforms in *Entamoeba histolytica* is not available, it can be safely presumed that it is one of the strategies for continuous and undisrupted cysteine production. The EhSAT3, which does not show feedback inhibition with final product Cys can be used for large scale industrial production of cysteine.

## Supporting Information

Figure S1 **Nucleotide sequencing of mutated EhSAT.** H208S-EhSAT1 and S208H-EhSAT3 mutations were confirmed by nucleotide sequencing. The mutated triplet codon has been highlighted.
(DOCX)

Figure S2 **Purification of proteins.** The purified protein after gel filtration were resolved on 12% SDS-PAGE and stained with coomassie blue. Lane 1 is protein marker, lane 2 is native EhSAT1 (34 kDa), lane 2 is H208S-EhSAT1, lane 3 is native EhSAT3 (37 kDa) and lane 4 is S208H-EhSAT3.
(DOCX)

Figure S3 **Modeling of** **active site co-ordination for H208S-EhSAT1, native EhSAT3 and S208H-EhSAT3.** A) According to the model H208S-EhSAT1, there are no interactions with Ser to bound amino acid (either Ser/Cys) bound in the active site and there is no structural change in orientation of His 223. B) EhSAT3 model was generated based on the experimentally determined coordinates of EhSAT1 (3P47). In the native EhSAT3, Ser 208 does not interact with bound Ser/Cys in the active site, C) while in mutant His 208-EhSAT3, the His residues forms hydrogen bond with carboxyl group of the bound amino acid.
(DOCX)

Figure S4 **Sequence alignment with watermelon SAT and** ***T. goesingense*** **SAT.** G277C mutation decreased the IC50 of the watermelon SAT by about 28 folds and the M280I mutation also had the same effect [24]. In EhSAT3 position equivalent to G277 is glutamate while that of M280 is already isoleucine. In EhSAT1 both these positions are occupied by Ile. Since EhSAT1 and EhSAT3 are differentially inhibited by cysteine, these residues may not have any effect over the cysteine inhibition. *T. goesingense* cytoplasmic SAT is feedback insensitive. Na and Salt identified P266 and A268 are responsible for making TgSAT insensitive to cysteine [25]. In EhSAT3 both the equivalent positions are occupied by glutamine and in EhSAT1, at 266 position there is serine while at 268 it is glutamine. These residues had nothing to be compared of and hence they might not be involved in the cysteine feedback inhibition in EhSATs.
(DOCX)
