# Supplementary material for: Single Residue Mutation in Active Site of Serine Acetyltransferase Isoform 3 from Entamoeba histolytica Assists in Partial Regaining of Feedback Inhibition by Cysteine
Source: PLoS One. 2013 Feb 21;8(2):e55932. doi: 10.1371/journal.pone.0055932 (PMC3578862; doi:10.1371/journal.pone.0055932)
Supplement: Figure S1 — Nucleotide sequencing of mutated EhSAT. H208S-EhSAT1 and S208H-EhSAT3 mutations were confirmed by nucleotide sequencing. The mutated triplet codon has been highlighted. (DOCX) [file pone.0055932.s001.docx]

Supplementary Figure S1. **Nucleotide sequencing of mutated EhSAT.** H208S-EhSAT1 and S208H-EhSAT3 mutations were confirmed by nucleotide sequencing. The mutated triplet codon has been highlighted.
