# Supplementary material for: Single Residue Mutation in Active Site of Serine Acetyltransferase Isoform 3 from Entamoeba histolytica Assists in Partial Regaining of Feedback Inhibition by Cysteine
Source: PLoS One. 2013 Feb 21;8(2):e55932. doi: 10.1371/journal.pone.0055932 (PMC3578862; doi:10.1371/journal.pone.0055932)
Supplement: Figure S2 — Purification of proteins. The purified protein after gel filtration were resolved on 12% SDS-PAGE and stained with coomassie blue. Lane 1 is protein marker, lane 2 is native EhSAT1 (34 kDa), lane 2 is H208S-EhSAT1, lane 3 is native EhSAT3 (37 kDa) and lane 4 is S208H-EhSAT3. (DOCX) [file pone.0055932.s002.docx]

Supplementary Figure S2. **Purification of proteins.** The purified protein after gel filtration were resolved on 12 % SDS-PAGE and stained with coomassie blue. Lane 1 is protein marker, lane 2 is native EhSAT1 (34 kDa), lane 2 is H208S-EhSAT1, lane 3 is native EhSAT3 (37 kDa) and lane 4 is S208H-EhSAT3.
