# Supplementary material for: Single Residue Mutation in Active Site of Serine Acetyltransferase Isoform 3 from Entamoeba histolytica Assists in Partial Regaining of Feedback Inhibition by Cysteine
Source: PLoS One. 2013 Feb 21;8(2):e55932. doi: 10.1371/journal.pone.0055932 (PMC3578862; doi:10.1371/journal.pone.0055932)
Supplement: Figure S3 — Modeling of active site co-ordination for H208S-EhSAT1, native EhSAT3 and S208H-EhSAT3. A) According to the model H208S-EhSAT1, there are no interactions with Ser to bound amino acid (either Ser/Cys) bound in the active site and there is no structural change in orientation of His 223. B) EhSAT3 model was generated based on the experimentally determined coordinates of EhSAT1 (3P47). In the native EhSAT3, Ser 208 does not interact with bound Ser/Cys in the active site, C) while in mutant His 208-EhSAT3, the His residues forms hydrogen bond with carboxyl group of the bound amino acid. (DOCX) [file pone.0055932.s003.docx]

Supplementary Figure S3. **Modeling of** **active site co-ordination for H208S-EhSAT1, native EhSAT3 and S208H-EhSAT3.** A) According to the model H208S-EhSAT1, there are no interactions with Ser to bound amino acid (either Ser / Cys) bound in the active site and there is no structural change in orientation of His 223. B) EhSAT3 model was generated based on the experimentally determined coordinates of EhSAT1 (3P47). In the native EhSAT3, Ser 208 does not interact with bound Ser/ Cys in the active site, C) while in mutant His 208-EhSAT3, the His residues forms hydrogen bond with carboxyl group of the bound amino acid.
