# Supplementary material for: Single Residue Mutation in Active Site of Serine Acetyltransferase Isoform 3 from Entamoeba histolytica Assists in Partial Regaining of Feedback Inhibition by Cysteine
Source: PLoS One. 2013 Feb 21;8(2):e55932. doi: 10.1371/journal.pone.0055932 (PMC3578862; doi:10.1371/journal.pone.0055932)
Supplement: Figure S4 — Sequence alignment with watermelon SAT and T. goesingense SAT. G277C mutation decreased the IC50 of the watermelon SAT by about 28 folds and the M280I mutation also had the same effect [24]. In EhSAT3 position equivalent to G277 is glutamate while that of M280 is already isoleucine. In EhSAT1 both these positions are occupied by Ile. Since EhSAT1 and EhSAT3 are differentially inhibited by cysteine, these residues may not have any effect over the cysteine inhibition. T. goesingense cytoplasmic SAT is feedback insensitive. Na and Salt identified P266 and A268 are responsible for making TgSAT insensitive to cysteine [25]. In EhSAT3 both the equivalent positions are occupied by glutamine and in EhSAT1, at 266 position there is serine while at 268 it is glutamine. These residues had nothing to be compared of and hence they might not be involved in the cysteine feedback inhibition in EhSATs. (DOCX) [file pone.0055932.s004.docx]

Supplementary Figure S4. **Sequence alignment with watermelon SAT and *T. goesingense* SAT.** G277C mutation decreased the IC50 of the watermelon SAT by about 28 folds and the M280I mutation also had the same effect [[24](#_ENREF_24)]. In EhSAT3 position equivalent to G277 is glutamate while that of M280 is already isoleucine. In EhSAT1 both these positions are occupied by Ile. Since EhSAT1 and EhSAT3 are differentially inhibited by cysteine, these residues may not have any effect over the cysteine inhibition. *T. goesingense* cytoplasmic SAT is feedback insensitive. Na and Salt identified P266 and A268 are responsible for making TgSAT insensitive to cysteine [[25](#_ENREF_25)]. In EhSAT3 both the equivalent positions are occupied by glutamine and in EhSAT1, at 266 position there is serine while at 268 it is glutamine. These residues had nothing to be compared of and hence they might not be involved in the cysteine feedback inhibition in EhSATs.
